# Increased functional connectivity following ingestion of dried bonito soup

**DOI:** 10.3389/fnut.2024.1354245

**Published:** 2024-04-03

**Authors:** Takatoshi Satake, Ai Taki, Kazuya Ouchi, Kazumi Kasahara, Tomokazu Tsurugizawa

**Affiliations:** ^1^Human Informatics and Interaction Research Institute, National Institute of Advanced Industrial Science and Technology (AIST), Tsukuba, Japan; ^2^Faculty of Engineering, Information and Systems, University of Tsukuba, Tsukuba, Japan

**Keywords:** functional MRI, dried bonito soup, human, functional connectivity, vermis

## Abstract

Soup, including dried bonito broth, is customarily consumed as an umami taste during meals in Japan. Previous functional magnetic resonance imaging (fMRI) studies have investigated neuronal activation following human exposure to carbohydrates and umami substances. However, neuronal activity following ingestion of dried bonito soup has not been investigated. Additionally, recent progress in fMRI has enabled us to investigate the functional connectivity between two anatomically separated regions, such as the default mode network. In this study, we first investigated the altered functional connectivity after ingesting dried bonito soup in healthy volunteers. Functional connectivity in several brain regions, including the connection between the vermis, part of the cerebellum, and bilateral central opercular cortex, was markedly increased after ingesting dried bonito soup, compared to the ingestion of hot water. Physiological scaling showed that satiety was substantially increased by ingesting hot water rather than dried bonito soup. These results indicate that increased functional connectivity reflects the post-ingestive information pathway of dried bonito soup.

## 1 Introduction

Soup that includes dried bonito broth provides an umami taste to Japanese foods (1). Dried bonito soup contains umami ingredients such as L-glutamate, inosine monophosphate (IMP), and amino acids, which exert anti-depressive (2) and anti-sympathetic nervous system effects (3). The ingestion of dried bonito soup ameliorates the aggressive behavior associated with perinatal dioxin exposure in children (4). In addition to behavioral and physiological studies, previous studies have investigated Fos expression in the rat brain after intragastric administration of dried bonito (5). Intragastric infusion of dried bonito increased Fos expression in the medial preoptic area, hypothalamus, and central nucleus of the amygdala, indicating that information regarding the ingested dried bonito was processed in the forebrain. Another study investigated the activity of the hypothalamus following infusion with amino acids, glucose, or lipid emulsions. The lateral hypothalamus was activated under amino acid and glucose conditions, but not under lipid emulsion condition (6).

Functional magnetic resonance imaging (fMRI) is a promising tool for noninvasive investigation of brain function. Previous studies have reported that blood oxygenation level dependent (BOLD) signal changes, which are closely linked to neuronal activation, are observed during taste (7, 8), smell (9), and food intake (10, 11). BOLD signals change in the hypothalamus after glucose intake in humans (11, 12) and rodents (13, 14). Additionally, the insular and opercular cortices are key regions for processing ingested food information (15). In contrast to glucose intake, few studies have investigated the brain response to umami substances such as L-glutamate and IMP in humans (16) and rodent models (10, 17).

Recent progress in fMRI has enabled the optimization of functional connectivity in the whole brain, which is derived from the synchronization of neuronal oscillations between anatomically-separated regions (18). Compared to local activation detected by task-based fMRI, functional connectivity includes more information on the composition of the wide brain network and complex information processing in the brain (19). Previous studies have shown that functional connectivity is related to food information processing (20) and cognitive function (21, 22). However, previous studies have focused on local activation after food intake (11) and no study has investigated the altered functional connectivity following the intake of dried bonito soup. In this study, we hypothesized that ingestion of the dried bonito soup changes the functional connectivity in the brain regions related to the processing of the information of ingested umami substance. We compared functional connectivity following the ingestion of dried bonito soup and hot water.

## 2 Materials and methods

### 2.1 Participants

Sixteen healthy volunteers (8 males and 8 females, mean age 35.9 ± 8.96 years) were recruited. All experimental procedures and protocols were approved by the Institutional Review Board of the National Institute of Advanced Industrial Science and Technology (AIST).

### 2.2 Visual analogue scale evaluation

The visual analogue scale (VAS) was used before each fMRI measurement according to the following three questions on hunger, satiety, and sleepiness (23). All the questions were asked in Japanese. (For example, if the hungriest state in your life is 100, how strong are your feelings of hunger now?) The participants answered the questions by placing a marker on the 140 mm line to indicate the score they felt was most appropriate for their current condition. The VAS responses were converted into hundredths of a percent and evaluated.

### 2.3 fMRI experiment

**Figure 1:**
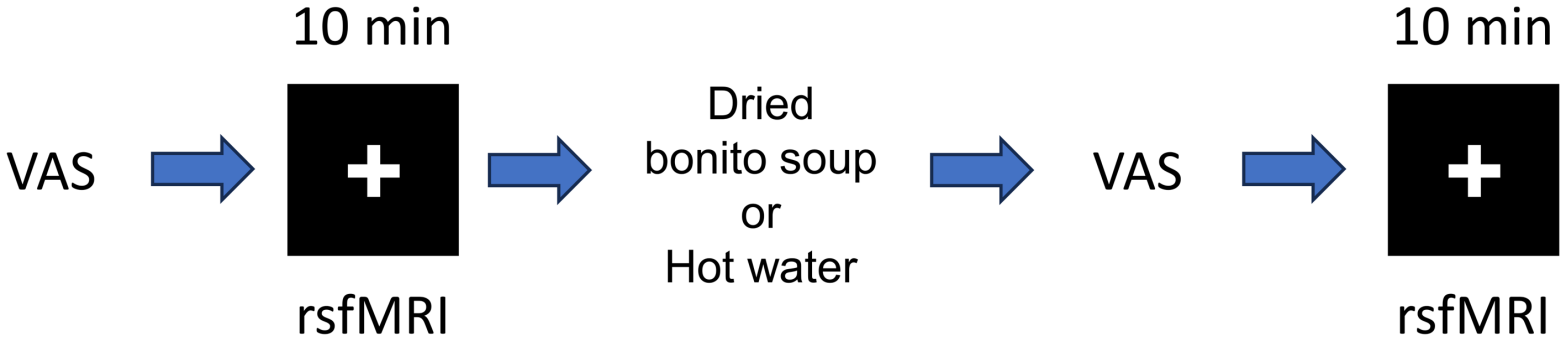
Schematic of the resting-state functional magnetic resonance imaging (fMRI). Schematic of resting-state fMRI and hot water or dried bonito soup ingestion. First, the participants answered the visual analogue scale (VAS) before first resting state fMRI. After ingestion of hot water or dried bonito soup, the participants answered the VAS again. The 2nd resting state fMRI was performed. Resting-state fMRI was performed for 10 min. The “+” mark was presented during the resting period.

The experimental setup is shown in Figure 1. The VAS evaluation was performed before MRI scanning. The MRI data were acquired using a 3 T MRI system with a 32-channel brain coil (Philips Healthcare, Best, Netherlands). Structural images were acquired using a magnetization-prepared rapid gradient echo (MPRAGE): TE/TR = 5.1/11 ms, flip angle = 8°, matrix = 368 × 315 × 44, resolution = 0.70 × 0.76 × 0.70 mm^3^/voxel. The fMRI was acquired to assess functional connectivity during the resting state with T2*-weighted gradient echo-planar imaging (EPI) with the following parameters: TE/TR = 30/1,500 ms, flip angle = 80°, matrix = 76 × 76 × 44, resolution = 2.5 × 2.5 × 2.5 mm^3^/voxel, 420 scans (in total 10 min and 30 s). The participants were instructed to watch a cross-mark on the screen. Resting-state functional MRI and structural imaging were performed before and after ingestion.

HONDASHI^®^ was used to create a dried bonito soup. HONDASHI^®^ soup is one of the products of the Ajinomoto Group (Ajinomoto Co., Inc., Kawasaki, Japan) and is made with monosodium L-glutamate (32.50%), disodium 5′inosinate (2.60%), disodium succinate (0.36%), non-iodized salt, lactose, sugar, dried bonito tuna powder, bonito extract, and yeast extract.^1^ The participants were asked to drink dried bonito soup, in which 3 g of dried bonito soup powder dissolved in 100 mL of hot water (approximately 40°C) or 100 mL hot water (~40°C) on a different day. Following the intake of dried bonito soup or hot water, the participants rested calmly for approximately 5 min, and the acquisition was started. The participants participated in the experiments on two separate days: one for dried bonito soup and the other for hot water. The sequence of dried bonito soup and hot water was randomly ordered for the counterbalanced design; that is, eight participants consumed dried bonito soup on the first day and eight participants consumed hot water on the first day. The gap between the first and second days was 160 ± 48 days.

### 2.4 fMRI analysis

#### 2.4.1 Preprocessing of fMRI data

All MRI data were preprocessed using statistical parametric mapping software SPM12 (Welfare Trust Center for Neuroimaging, United Kingdom) to perform preprocessing steps. Preprocessing included slice timing, motion correction by realignment, and normalization of structural and functional data into a standard MNI space. Subsequently, the functional images were smoothed with an FWHM kernel of 8 × 8 × 8 mm^3^.

#### 2.4.2 Functional connectivity analysis

The head motion was checked with Artifact Detection Tools (ART).^2^ The framewise displacement (FD), which is effective on the fMRI signals (24), was calculated using this tool. The outliers were detected when maximum FD >1.5 mm or 1.5° in all participants (25). Functional connectivity was then analyzed using the CONN toolbox.^3^ The preprocessed fMRI data were then detrended. The mean signals in the ventricles and white matter, and six motion parameters of the head (translational and rotational motions) were regressed from the time series of each voxel to reduce the contribution of physiological noise, such as respiration and head movement. Slow periodic fluctuations were extracted using a bandpass filter (0.008–0.08 Hz). The regions of interest (ROI) were defined using the 132 anatomical regions provided by default in the CONN toolbox. The change in functional connectivity was generated for each condition (pre- and post-intake) as the product of the ROI time series multiplied by intake, and the beta weight was calculated for all ROIs. At the group level, random effects analysis was used across participants, and a *t*-test was conducted to compare ROI-based connectivity in each condition (hot water and dried bonito soup). The statistical significance of ROI–ROI connectivity was assessed using an uncorrected threshold of *p* < 0.001. Seed-based functional connectivity analysis was performed based on the results of the ROI–ROI functional connectivity, and the seeds were determined to be the vermis 6 (Ver 6), cerebellum, and bilateral central operculum (CO). The significance of seed-based functional connectivity was thresholded at *p* < 0.05, using family-wise error correction.

### 2.5 Statistics of VAS score

A paired *t*-test between the pre- and post-ingestion in each question and condition (dried bonito or hot water) was performed on the VAS scores following a two-way repeated measures analysis of variance. We conducted a paired *t*-test to analyze the change in the VAS ratio between pre- and post-ingestion for each condition.

## 3 Results

### 3.1 Physiological parameters

We conducted a two-factor repeated-measures analysis of variance on the conditions, and pre- and post-ingestion values were different (*F* (1,15) = 9.525, *p* = 0.008). We did not find any differences in the hot water and dried bonito soup conditions (*F* (1,15) > 0.000, *p* = 0.998) or interaction effects (*F* (1,15) = 2.145, *p* = 0.164).

**Figure 2:**
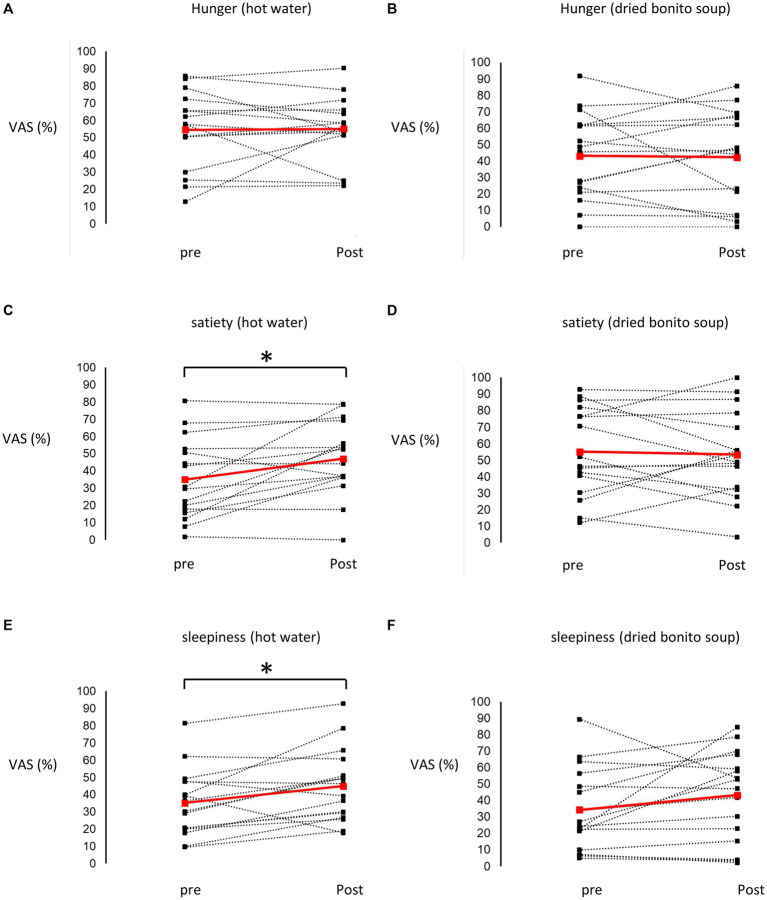
Results of the visual analogue scale (VAS) method. **(A,B)** The VAS of hunger before and after ingesting **(A)** hot water and **(B)** dried bonito soup. **(C,D)** The VAS of satiety before and after ingesting **(C)** hot water and **(D)** dried bonito soup. **(E,F)** The VAS of sleepiness before and after ingesting **(E)** hot water and **(F)** dried bonito soup. The red straight line shows the average change, and the black dashed line shows the change from pre-ingestion to post-ingestion of hot water or dried bonito soup in each participant. Statistical results are indicated using significant difference (^*^p < 0.05 between pre- and post- by paired *t*-test).

**Figure 3:**
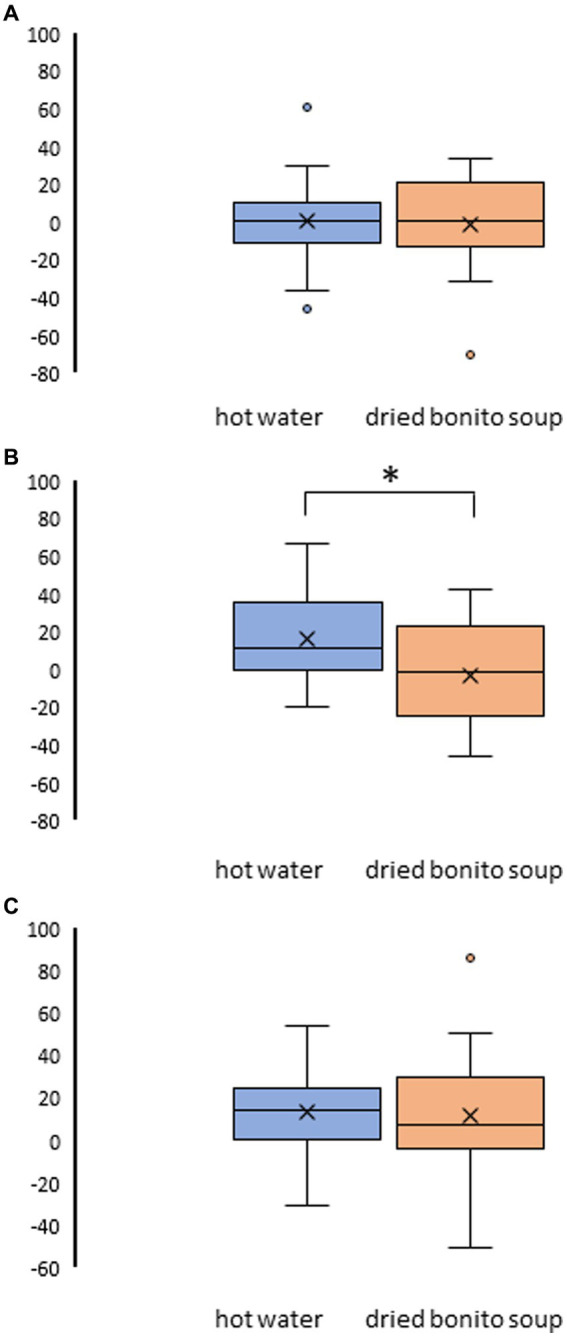
Result of the visual analogue scale (VAS) method in pre- and post-ingestion changes. **(A)** The change in VAS on hunger pre- and post-ingestion. **(B)** The change in VAS on satiety pre- and post-ingestion. **(C)** The change in VAS on sleepiness pre- and post-ingestion. The boxplots illustrate the comparison between hot water and dried bonito soup. The blue boxplot represents hot water and the orange boxplot represents dried bonito soup. The results indicate a statistical significance (^*^p < 0.05 between pre and post using paired *t*-test).

After ingesting the dried bonito soup or hot water, hunger, satiety, and sleepiness were assessed (Figure 2). Hunger was not substantially altered by the ingestion of hot water or dried bonito soup (Figures 2A,B). The VAS scores for satiety and sleepiness increased following hot water ingestion (*p* < 0.05) (Figures 2C,E). However, ingestion of dried bonito did not markedly alter satiety or sleepiness (Figures 2D,F). Sleepiness was similar for both hot water and dried bonito soup and increased after ingestion (Figures 2E,F). A *t*-test was conducted to examine whether there was a difference in the changes in VAS before and after ingestion between the groups. The results of a paired *t*-test showed that the change was marked only in satiety (*p* = 0.01), with a greater increase in satiety for hot water than for dried bonito soup (Figure 3).

The averaged FDs were 0.119 ± 0.006 mm for dried bonito pre-ingestion, 0.117 ± 0.006 mm for dried bonito post-ingestion, 0.116 ± 0.007 mm for hot water pre-ingestion, and 0.113 ± 0.007 mm for hot water post-ingestion. There was no significant difference in FDs among the conditions (*p* > 0.05 by two-way repeated ANOVA). These results indicate that ingestion of dried bonito soup or hot water did not affect the head motion during fMRI scanning.

### 3.2 Increased functional connectivity following ingestion of dried bonito soup

**Figure 4:**
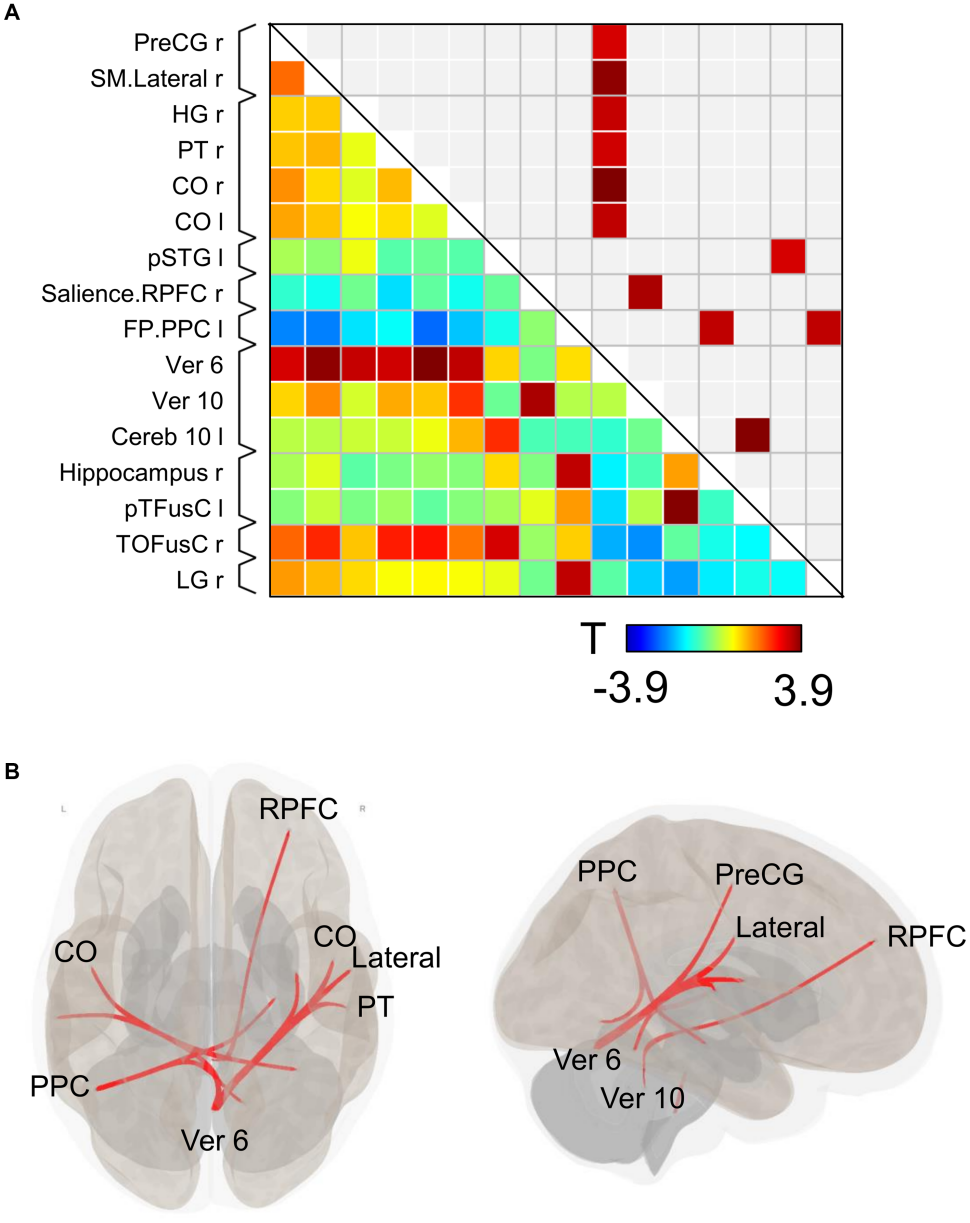
Connectivity matrix and glass brain. **(A)** Represents the functional connectivity of the regions of interest. The elements indicate the strength of connectivity between regions. In **(B)**, regions with strong connectivity are spatially represented by a glass brain. PreCG, the precentral gyrus; SM lateral, lateral sensorimotor network; HG, Heschl’s gyrus; PT, planum temporale; CO, central opercular cortex; pSTG, posterior part of the superior temporal gyrus; RPFC, right rostral prefrontal cortex; PPC, posterior parietal cortex; Ver 6, vermis 6; Ver 10, vermis 10; Cereb 10, cerebellum 10; pTFusC, posterior part of temporal fusiform cortex; TOFusC, temporal occipital fusiform cortex; LG, lingual gyrus.

Substantial changes in functional connectivity following the ingestion of dried bonito soup were compared with those following hot water ingestion (Figure 4A). Overall, functional connectivity with Ver 6 was increased in many brain regions, such as the motor cortex [right precentral gyrus (PreCG) and lateral sensorimotor network], temporal lobe [right Heschl’s gyrus (HG), right planum temporale (PT), and bilateral central opercular cortex (CO)]. Functional connectivity between the vermis 10 and the right rostral prefrontal cortex (RPFC), which is part of the salience network, increased. Functional connectivity in the left posterior parietal cortex (PPC), which is part of the frontoparietal network, with the right hippocampus and right lingual gyrus increased. Functional connectivity increased between the left posterior part of the superior temporal gyrus (pSTG) and the right temporal occipital fusiform cortex (TOFusC). The spatial distribution of functional connectivity showed that bilateral increases were induced by Ver 6 (Figure 4B).

### 3.3 Seed-based functional connectivity following ingestion of dried bonito soup

**Figure 5:**
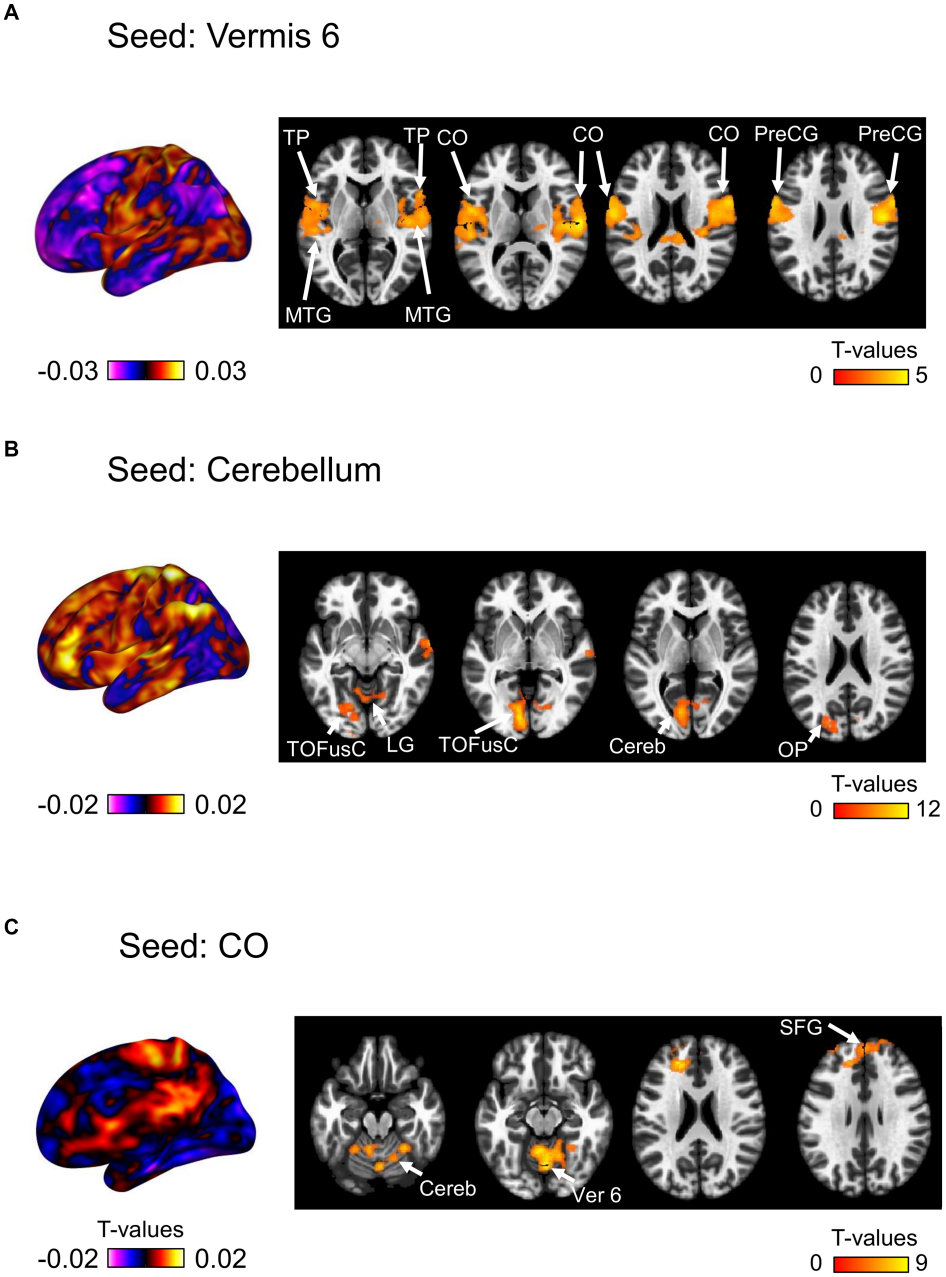
Seed-based functional connectivity. This represents the functional connectivity of each of the three regions as seeds. **(A)** Functional connectivity of vermis 6 as a seed, **(B)** cerebellum, and **(C)** central opercular cortex. The color bar indicates the *t*-value.

We then investigated the changes in seed-based functional connectivity following the ingestion of dried bonito soup compared to the ingestion of hot water (Figure 5). Functional connectivity between Ver 6 and the bilateral CO, PreCG, TP, and middle temporal gyrus (MTG) increased substantially (Figure 5A). When the cerebellum was selected as the seed, functional connectivity with the left TOFusC and left occipital pole (OP) increased (Figure 4B). Increased functional connectivity with the bilateral CO was also observed (Figure 4C). Functional connectivity with the CO, superior frontal gyrus (SFG), cerebellum, and Ver 6 increased following the ingestion of dried bonito soup.

## 4 Discussion

In this study, we aimed to investigate the altered functional connectivity following the ingestion of dried bonito soup. Increased functional connectivity after ingesting dried bonito soup compared to hot water intake was successfully demonstrated. Functional connectivity between the vermis and several regions of the temporal lobe increased substantially. Additionally, the functional connectivity with parts of the sensorimotor and salience networks increased. Previous studies have focused on the local activation of BOLD signaling changes after ingesting nutrients such as carbohydrates and amino acids. This approach enabled the investigation of the altered neuronal activation after nutrient ingestion. However, it is impossible to investigate altered functional connectivity, which is defined as the synchronization of BOLD signal fluctuations between separate regions and is related to the processing of ingested information. This study clearly demonstrates that functional connectivity changes after the ingestion of dried bonito soup. Brain regions are key to processing ingested food information through the afferent vagus nerve.

### 4.1 Role of the vermis-temporal lobe network

Functional connectivity between Ver 6 and the temporal lobes, including the bilateral CO, right HG, and right PT, was markedly increased. The operculum and vermis are involved in processing information regarding food-induced odors, such as chocolate (26). The vermis, a part of the cerebellum, is involved in the bottom-up appetitive network and plays a prominent role in feeding behavior, particularly in the drive to approach appetizing stimuli. Women with anorexia nervosa have reduced appetite and reduced responses to food images when explicitly thinking about eating food, as shown in the images. These subtypes are differentiated by increased or reduced activation in regions associated with appetitive and somatosensory impulsive responses, such as the dorsal striatum, insular cortex, and cerebellar vermis (27).

The vermis is essential for connecting the visceral organs and brain through the vagus nerve (28). Vagus nerve stimulation activates the vermis and CO (29). Rebollo et al. showed marked phase coupling between the electrogastrogram and resting-state BOLD time series in 12 nodes, called the “gastric network”; CO and Ver 6 are included in the “gastric network” (30). Ingested umami substances such as L-glutamate and IMP increase vagal nerve activity (31). These results indicate that increased functional connectivity between Ver 6 and the bilateral CO may be related to gut–brain interactions. The insular - opercular cortex is associated with spatiotemporal information regarding food intake. During meal consumption, time-locked high-frequency broadband activity at the time of food intake depends on the food types and is associated with cue-specific activity (32). The consumption of palatable foods results in greater activation of the frontal cortex and operculum/insula than the consumption of unpalatable foods (33). These results support the increased functional connectivity between the bilateral CO and the superior frontal gyrus due to seed-based functional connectivity.

### 4.2 Comparison between the local signal change and connectivity

Previous studies have investigated the changes in BOLD signals following the ingestion of nutrients. In contrast, the present study investigated altered functional connectivity. BOLD signal changes reflect changes in neuronal activity in local regions (34). The BOLD signal in the hypothalamus decreases substantially after ingestion of glucose (11, 35). However, compared with glucose studies, there is insufficient evidence for the neuronal imaging of umami substance ingestion. Several studies have reported that the intragastric infusion of nutrients such as umami substances and carbohydrates induces BOLD signal changes (10, 13, 14, 36). These results showed that the increased BOLD signal changes were prolonged for more than 10 min, indicating that ingested nutrients affect resting-state fMRI 5–20 min after ingestion. In contrast to BOLD signal changes, functional connectivity reflects the synchronicity of BOLD signal fluctuations between anatomically separated regions. This BOLD fluctuation is related to cognitive function (37); psychiatric diseases induce abnormal functional connectivity (38). However, no study has investigated functional connectivity after food intake.

In this study, we clearly showed altered functional connectivity related to the gut-brain axis and food intake. To our knowledge, this is the first study to demonstrate the relationship between functional connectivity and food intake in humans. A rat study revealed that functional connectivity is decreased by sucrose intake (39). The *ad libitum*-fed rats showed a trend toward higher functional connectivity than food-restricted rats. Functional connectivity is affected by the nutritional status of the body. Functional connectivity from the posterior to the anterior insula is strengthened by hunger compared to satiety, and glucose intake alters functional connectivity (40). In a mouse study, food deprivation increased the functional connectivity between the audiovisual cortex, hippocampus, and retrosplenial cortex (41). These studies indicate that functional connectivity is influenced by the nutrient state and ingested food. Therefore, the altered functional connectivity due to dried bonito soup intake is reasonable and provides insights into the physiological significance of dried bonito soup in Japanese cuisine. However, dried bonito soup contains several food ingredients, such as salt, and more precise studies using L-glutamate or IMP should be performed in the future. The objective of this study was to investigate the altered functional connectivity following the ingestion of dried bonito soup. This may have been caused by taste, ingestion, and post-ingestive effects. In the future, the effects of taste, smell, and post-ingestion of dried bonito soup should be investigated separately to understand the mechanisms of altered functional connectivity.

### 4.3 The limitations of this study

We investigated the functional MRI (fMRI) data of 16 participants. Although previous studies showed good quality results and the results of increased BOLD areas were replicable with less than 20 participants (15, 42, 43), there was limited statistical power in this study due to the small sample size (44). Marek et al. (44) used the big data (50,000 individuals) to estimate the sample size for improving replication rates and decreasing effect size inflation. Other studies estimate the sample size for reliable reproducibility and 40–300 individuals are required (45, 46). Future studies should use larger sample sizes to assess the reproducibility of results.

In the present study, we selected the VAS method to measure the participants’ feelings of hunger, and sleepiness because it has been used in the previous fMRI study (45, 47, 48). Although there is no difference in the resolving power between VAS and general labeled magnitude scale (gLMS) (49), previous studies have used the gLMS to measure the perceptual intensity, such as taste, smell, and hunger (50–52). We will compare the VAS and gLMS to measure the physiological state.

In the present study, we did not calculate the brain response to the intake of dried bonito soup because the intake of dried bonito soup or hot water was performed outside of the MRI bore to avoid the motion artifacts in ingestion. Therefore, we could not directly compare the relationship between local neuronal activation and altered functional connectivity with dried bonito soup. Future studies should attempt to compare them using fMRI by conducting ingestion in the MRI bore.

## 5 Conclusion

In conclusion, our study showed that dried bonito soup ingestion increased the functional connectivity in the regions involved in the information processing of ingested food, such as the vermis, central opercular cortex, a part of sensorimotor and temporal lobes. These results indicate that functional connectivity can be a marker of ingested food information in the brain.

## Data availability statement

The original contributions presented in the study are included in the article/supplementary material, further inquiries can be directed to the corresponding author.

## Ethics statement

The studies involving humans were approved by Institutional Review Board of the National Institute of Advanced Industrial Science and Technology. The studies were conducted in accordance with the local legislation and institutional requirements. The participants provided their written informed consent to participate in this study.

## Author contributions

TS: Data curation, Formal analysis, Investigation, Visualization, Writing – original draft. AT: Data curation, Investigation, Writing – original draft. KO: Data curation, Investigation, Writing – review & editing. KK: Writing – review & editing. TT: Conceptualization, Data curation, Formal analysis, Funding acquisition, Investigation, Methodology, Project administration, Supervision, Validation, Visualization, Writing – original draft, Writing – review & editing.

## Footnotes

^1^ https://www.ajinomoto.co.th/en/our-product/food-service/hon-dashi

^2^ https://www.nitrc.org/projects/artifact_detect/

^3^ www.conn-toolbox.org
